# Cemiplimab and Cutaneous Squamous Cell Carcinoma: From Bench to Bedside

**DOI:** 10.1016/j.jpra.2022.06.003

**Published:** 2022-06-23

**Authors:** D.T. Goodman

**Affiliations:** Department of Plastic Surgery, Cork University Hospital, Wilton, Co Cork, Ireland

**Keywords:** Cemiplimab, Translational medicine, Squamous cell carcinoma, PD-1 inhibitor

## Abstract

Non-melanoma skin cancers (NMSCs) are the most common cancer in fair-skinned individuals with basal cell carcinoma (BCC) and squamous cell carcinoma (SCC) being the most common subtype. While BCC has historically been the most common NMSC, SCC is increasing in incidence relative to BCC. SCC has a very poor prognosis with advanced local infiltration or when it achieves a metastatic state with around 50% of patients with locally advanced disease relapsing with an average overall survival of 10–13 months for patients with recurrent or metastatic disease.

The pathogenesis of cutaneous SCC (cSCC) is multifactorial, and many studies have also described in detail the strong link between tumour apoptosis, DNA repair mechanism deficiencies, and developing cSCC. Patients with TP53 mutations are more susceptible to develop cSCC, thus highlighting the importance of cell cycle regulation and also pointing towards the potential therapeutic targets within.

This review illustrates the role of the programmed death receptor-1 (PD-1) inhibitor cemiplimab in treating advanced and metastatic cSCC not suitable to surgical excision and describes its development in the context of the translational research paradigm from preclinical studies to its licenced implementation in clinical care and beyond.

## Introduction

Non-melanoma skin cancers (NMSCs) are the most common cancer in fair-skinned individuals with basal cell carcinoma (BCC) and squamous cell carcinoma (SCC) being the most common subtypes.^1^ While BCC has historically been the most common NMSC, SCC is increasing in incidence relative to BCC. Previous BCC:SCC ratio of 4:1 is now estimated to be 2.5:1 in Australia, while in the USA, an overall increase of 100% of cases has been reported from 1992 to 2012.^2^ The risk factors for cutaneous SCC (cSCC) include cumulative sun exposure, having fair skin, immunosuppression, and advanced age^3^ with surgical excision being the mainstay of treatment in 95% of cases.^4,^^5^ However, SCC has a very poor prognosis with advanced local infiltration or when it achieves a metastatic state^6,^^7^ with around 50% of patients with locally advanced disease relapsing with an average overall survival of 10–13 months for patients with recurrent or metastatic disease.^8^

The pathogenesis of cSCC is multifactorial. While the link between UV radiation exposure and NMSC is well known^1^, many studies have also described in detail the link between a compromised immune system and cSCC development, specifically in those patients who have received solid organ transplants, a cohort in which cSCC occurs 65–250 times more frequently.^9^ The increased incidence rate of cSCC in patients with HIV infection and non-Hodgkin's lymphoma is also well documented in the literature.^10,^^11^

There is a strong link between tumour apoptosis, DNA repair mechanism deficiencies, and the development of cSCC. Patients with TP53 mutations^12^ and those with impaired DNA repair abilities, such as xeroderma pigmentosum and Muir–Torre syndrome, have been shown to be more susceptible to cSCC development.^13^ These discoveries highlight the importance of cell cycle regulation and point towards the potential therapeutic targets within. The aim of this review is to chronicle the development and role of programmed cell death receptor 1 (PD-1) inhibitor cemiplimab in the setting of cSCC.

## Methodology

This narrative review describing the development and utilisation of a novel therapeutic agent in treating cSCC was conducted using MEDLINE and PubMed in January 2022 to identify animal models, pre-clinical studies, and human clinical trials to chronicle the development of cemiplimab.

A comprehensive search was performed using the following keywords: ‘cemiplimab’ [All Fields] AND ‘REGN2810’ [All Fields] AND ‘squamous cell carcinoma’ [All Fields] AND ‘clinical trials’ [All Fields].

One researcher (DG) performed the search independently. Papers were interrogated for the latest trials in relation to developments and outcomes associated with the use of cemiplimab in relation to cSCC. All searches were conducted in January 2022. Only full publications in English were considered, and abstracts were excluded. PRISMA guidelines were not adhered to as this narrative review focused on summarising the development of cemiplimab from a translational medicine perspective and providing an overview of the topic.

### Target Discovery

The concept of immunoediting introduced by Dunn et al.^14^ described the immune system's influence on neoplastic progression and the intricate relationship between all stages of tumour progression and the host response, which allows tumour cells to evade recognition by immune effector cells. For many years, researchers sought to stimulate an anti-tumour host immune response and initially the inhibitory pathways that control the function of this t-cells response stood in the way of eliciting the desired effect.

Both cytotoxic T lymphocyte-associated antigen 4 (CTLA-4) and programmed cell death receptor 1 (PD-1) inhibit lymphocyte activation, while t-cell antigen receptors (TCR) such as CD28 and inducible T-cell co-stimulator (ICOS) transduce signals that activate T cells.^15^ It is this balance between positive and negative regulation of T-lymphocyte proliferation which is essential to the acquired immune response while maintaining immunological tolerance and preventing autoimmunity.^15^ PD-1 has two ligands: PD-L1 and PD-L2. PD-L1 is the primary ligand which is upregulated in solid tumours and on cells within the tumour microenvironment which respond to inflammatory stimuli.^16^. Antagonism of the PD-1/PD-L1 interaction enhances the immune response in vitro and moderates preclinical antitumour activity making PD-L1 a promising target for cancer immunotherapy.^16^

### In vitro/In vivo Pre-clinical Studies

Amarnath et al. described in 2011 the relationship between T helper type 1 (Th1) cells, host autoimmunity, and graft-versus-host disease (GvHD) after organ transplantation using murine models and transfecting small interfering RNA in cultured cells.^17^ The in vivo elements of this study showed how the overexpression of PD-L1 led to the conversion of Th1 cells to regulatory T cells which prevented xenogenic GvHD, whereas blocking PD-1 expression in Th1 cells restored their ability to mediate GvHD.^17^ They surmised that blocking the conversion of Th1 to regulatory T cells using PD1 antagonists could provide a new clinical translational approach to enhance T cell immunity to cancer and infection.^17^

Burova et al. subsequently described the preclinical characterisation of REGN2810, which became to be known as cemiplimab, a high-affinity monoclonal IgG4 anti-PD-1 antibody that antagonises the interactions between PD-1 and its ligands ^18^. In cell-based assays, REGN2810 was shown to block the PD-1/PD-L1 interaction, thus increasing TCR signalling and proliferation of primary activated human T cells.^18^ Using human PD-1 knock-in mice to study the in vivo efficacy of REGN2810, this group demonstrated that MC38 murine tumour growth was inhibited while also providing a measurable survival benefit.

### Phase 1 Trials

Migden et al. conducted a phase 1 trial of cemiplimab.^19^ This was an open-label, non-randomised multicentre study which included 26 immunocompetent adult patients who had advanced cSCC and were treated with three different doses of cemiplimab administered intravenously every two weeks. The response rate was characterised according to the standardised Response Evaluation Criteria in Solid Tumour, version 1.1. This study showed a 50% objective response rate with a median response time of 2.3 months. The median follow-up was 11.0 months. The exclusion criteria for this trial included those who had concurrent cancer, recipients of solid organ transplant, immunocompromised, or those previously treated with a PD-1 inhibitor. A total of 27% of patients reported feeling fatigued while 15% of patients reported adverse effects, such as constipation, diarrhoea, nausea, decreased appetite, urinary tract infections, and electrolyte disturbances. This adverse effect profile is broadly similar to those seen with other PD-1 inhibitors.^19^

Another study by Owonikoko et al. followed on from the phase 1 trial from Migden et al. with longer follow-up efficacy and safety data. This study enrolled 26 patients and again showed an overall response rate of 50% and that the duration of response exceeded 6 months in 9 patients and 12 months in 5 patients with a similar rate of adverse events described by Migden et al., thereby confirming a positive risk/benefit profile for cemiplimab treating advanced cSCC.^20^

### Phase II/III Trials

In 2018, Migden et al. published the results from his group's phase 2 study describing the efficacy of cemiplimab in metastatic cSCC in immunocompetent patients not amenable to curative surgery or curative radiation.^19^ This multicentre, non-randomised control trial enrolled 59 patients and again treated them with intravenous cemiplimab at 3 mg/kg every 2 weeks. A response was observed in 47% of patients with 57% of responders showing a duration of response that exceeded 6 months and 82% of these patients continuing to have a response after the time of data cut off.^19^ The exclusion criteria of this phase 2 trial were the same as the aforementioned phase 1 study from Migden et al. The authors reported that the estimated probability of progression-free survival at 12 months was 81%. Adverse events in the metastatic-disease cohort occurred in 15% of patient and included diarrhoea, fatigue, nausea, constipation, and rash.^19^

### FDA/EMA Approval

In 2018, the US Food and Drug Administration approved cemiplimab-rwlc for patients with metastatic cSCC or locally advanced cSCC who are not candidates for surgery or curative radiation with a dosing schedule of 350 mg IV infusion over 30 min every 3 weeks.^5,^^21^ Cemiplimab-rwlc has since been approved for the same clinical indications by the European Medicines Agency, Health Canada, and the UK National Institute for Health and Care Excellence.^22^

### Post-approval Developments

Following FDA approval, Rischin et al. published a phase 2 study of cemiplimab in patients with metastatic cSCC focusing on a primary analysis of fixed dosing 350 mg IV every 3 weeks and also looked at long-term outcomes of the 3 mg/kg IV every 2 weeks dosing regimen. This multicentre non-randomised control trial enrolled 56 patients treated with fixed dosing for a median duration of 8.1 months. Exclusion criteria included the immunosuppressed, solid organ transplant recipients, prior treatment with PD-1/PD-L1 antagonists, concurrent cancers, or haematological cancers. The objective response rate was 41.1% illustrating substantial antitumour activity while the duration of response at 8 months was 95% among responding patients.^23^ A separate arm of the same study looked at 11-month follow-up from the cohort described by Migden et al.^19^ A total of 59 patients were treated with cemiplimab at 3 mg/kg Iv every 2 weeks. The response rate increased to 49.2% and duration of response was estimated at 88.9% at 12 months among responding patients.^23^ However, 71.3% of participants in both treatment arms reported treatment-related adverse events (TRAE) with the most common being fatigue (13.0%), maculopapular rash (11.3%), hypothyroid (10.4), and diarrhoea (9.6%).^23^

Currently, there in an active phase 3 trial studying cemiplimab's effects as an adjuvant versus placebo after surgery and radiation therapy in patients with high-risk cSCC which is due to publish its first results in October 2023^24^ This randomised, placebo-controlled, double-blind, multicentre trial has enrolled 75 participants to date. The primary outcome measure is disease-free survival at 54 months while secondary outcomes include overall survival, the timeframe of regional recurrence and freedom from distal recurrence, occurrence of second primary cSCC, and adverse effects.

### Current Status

Regeneron is now recruiting 350 patients for a phase 4 trial, for their CASE (cemiplimab-rwlc Survivorship and Epidemiology) study which will be a multicentre, prospective, non-interventional cohort study of adult patients with advanced cSCC receiving cemiplimab.^22^ This study will report on outcomes and long-term effectiveness of cemiplimab, progression patterns of patients participating in the study, and patient-related outcomes. The authors of this study describe their purpose as bridging the evidence gap between clinical trials and cemiplimab use in the real-world setting.^22^

In addition to being licenced for treating cSCC, there have been many developments in its role in treating other malignancies. Sezar et al. have recently published results from a phase 3 multicentre, open-label, randomised controlled trial comparing cemiplimab to platinum-based chemotherapies in patients with metastatic non-small cell lung cancer with PD-L1 of at least 50%.^25^ This trial (EMPOWER-Lung 1) recruited 710 adult patients, with one group treated with 350 mg IV every 3 weeks, and the other groups treated with platinum-doublet chemotherapy. Patients who had smoked less than 100 cigarettes in their lifetime were excluded. In the group treated with cemiplimab, the median overall survival was not reached compared to 14.2 months in those treated with chemotherapy.^25^ Median progression-free survival with cemiplimab was 8.2 months versus 5.7 months with chemotherapy. TRAE occurred in 57% of patients receiving cemiplimab versus 89% in those receiving chemotherapy with grade 3-4 TRAE incidence of 12% in the cemiplimab group versus 37% in the chemotherapy group.

Another phase 3 trial studying the effects of cemiplimab as a monotherapy in advanced cervical cancer (adenocarcinoma and SCC variants) has concluded ahead of its estimated study completion date in July 2023 following promising initial results. This multicentre, open-label randomised control trial with 590 participants demonstrated a 31% reduced risk of death in advanced cervical cancer compared to treatment with chemotherapy.^26^

There also are many other trials currently in their early stages investigating the role of cemiplimab in treating glioblastoma multiforme, BCC, and renal cell carcinoma.^27^, ^28^, ^29^

This review has illustrated the role of the PD-1 inhibitor cemiplimab in treating advanced and metastatic cSCC not suitable to surgical excision and has described its development in the context of the translational research paradigm from preclinical studies to its licenced implementation in clinical care. While we do not have the complete data to describe its T4 translation, we have seen that its efficacy and favourable adverse effect profile make cemiplimab a success story in treating advanced and metastatic cSCC.

## Funding

None

## Ethical approval

Not required

## Conflicts of interest

None declared

## References

[1] Lomas A, Leonardi-Bee J, Bath-Hextall F (2012). A systematic review of worldwide incidence of nonmelanoma skin cancer. Br J Dermatol.

[2] Rogers HW, Weinstock MA, Feldman SR, Coldiron BM (2015). Incidence Estimate of Nonmelanoma Skin Cancer (Keratinocyte Carcinomas) in the U.S. Population, 2012. JAMA Dermatol.

[3] Stratigos A, Garbe C, Lebbe C, Malvehy J, del Marmol V, Pehamberger H, Peris K, Becker JC, Zalaudek I, Saiag P, Middleton MR, Bastholt L, Testori A, Grob JJ (2015). Diagnosis and treatment of invasive squamous cell carcinoma of the skin: European consensus-based interdisciplinary guideline. Eur J Cancer.

[4] Kauvar AN, Arpey CJ, Hruza G, Olbricht SM, Bennett R, Mahmoud BH (2015). Consensus for Nonmelanoma Skin Cancer Treatment, Part II: Squamous Cell Carcinoma, Including a Cost Analysis of Treatment Methods. Dermatol Surg.

[5] Administration FaD (2019) FDA approves cemiplimab-rwlc for metastatic or locally advanced cutaneous squamous cell carcinoma. https://www.fda.gov/drugs/drug-approvals-and-databases/fda-approves-cemiplimab-rwlc-metastatic-or-locally-advanced-cutaneous-squamous-cell-carcinoma. Accessed April 14, 2021

[6] Hillen U, Leiter U, Haase S, Kaufmann R, Becker J, Gutzmer R, Terheyden P, Krause-Bergmann A, Schulze HJ, Hassel J, Lahner N, Wollina U, Ziller F, Utikal J, Hafner C, Ulrich J, Machens HG, Weishaupt C, Hauschild A, Mohr P, Pföhler C, Maurer J, Wolff P, Windemuth-Kieselbach C, Schadendorf D, Livingstone E (2018). Advanced cutaneous squamous cell carcinoma: A retrospective analysis of patient profiles and treatment patterns-Results of a non-interventional study of the DeCOG. Eur J Cancer.

[7] Brunner M, Veness MJ, Ch'ng S, Elliott M, Clark JR (2013). Distant metastases from cutaneous squamous cell carcinoma–analysis of AJCC stage IV. Head Neck.

[8] Vathiotis IA, Johnson JM, Argiris A (2021). Enhancing programmed cell death protein 1 axis inhibition in head and neck squamous cell carcinoma: Combination immunotherapy. Cancer Treat Rev.

[9] Mittal A, Colegio OR (2017). Skin Cancers in Organ Transplant Recipients. Am J Transplant.

[10] Silverberg MJ, Leyden W, Warton EM, Quesenberry CP, Engels EA, Asgari MM (2013). HIV infection status, immunodeficiency, and the incidence of non-melanoma skin cancer. J Natl Cancer Inst.

[11] Brewer JD, Shanafelt TD, Khezri F, Sosa Seda IM, Zubair AS, Baum CL, Arpey CJ, Cerhan JR, Call TG, Roenigk RK, Smith CY, Weaver AL, Otley CC (2015). Increased incidence and recurrence rates of nonmelanoma skin cancer in patients with non-Hodgkin lymphoma: a Rochester Epidemiology Project population-based study in Minnesota. J Am Acad Dermatol.

[12] Wikonkal NM, Brash DE (1999). Ultraviolet radiation induced signature mutations in photocarcinogenesis. J Investig Dermatol Symp Proc.

[13] Schierbeck J, Vestergaard T, Bygum A (2019). Skin Cancer Associated Genodermatoses: A Literature Review. Acta Derm Venereol.

[14] Dunn GP, Old LJ, Schreiber RD (2004). The three Es of cancer immunoediting. Annu Rev Immunol.

[15] Parry RV, Chemnitz JM, Frauwirth KA, Lanfranco AR, Braunstein I, Kobayashi SV, Linsley PS, Thompson CB, Riley JL (2005). CTLA-4 and PD-1 receptors inhibit T-cell activation by distinct mechanisms. Mol Cell Biol.

[16] Brahmer JR, Tykodi SS, Chow LQ, Hwu WJ, Topalian SL, Hwu P, Drake CG, Camacho LH, Kauh J, Odunsi K, Pitot HC, Hamid O, Bhatia S, Martins R, Eaton K, Chen S, Salay TM, Alaparthy S, Grosso JF, Korman AJ, Parker SM, Agrawal S, Goldberg SM, Pardoll DM, Gupta A, Wigginton JM (2012). Safety and activity of anti-PD-L1 antibody in patients with advanced cancer. N Engl J Med.

[17] Amarnath S, Mangus CW, Wang JC, Wei F, He A, Kapoor V, Foley JE, Massey PR, Felizardo TC, Riley JL, Levine BL, June CH, Medin JA, Fowler DH (2011). The PDL1-PD1 axis converts human TH1 cells into regulatory T cells. Sci Transl Med.

[18] Burova E, Hermann A, Waite J, Potocky T, Lai V, Hong S, Liu M, Allbritton O, Woodruff A, Wu Q, D'Orvilliers A, Garnova E, Rafique A, Poueymirou W, Martin J, Huang T, Skokos D, Kantrowitz J, Popke J, Mohrs M, MacDonald D, Ioffe E, Olson W, Lowy I, Murphy A, Thurston G (2017). Characterization of the Anti-PD-1 Antibody REGN2810 and Its Antitumor Activity in Human PD-1 Knock-In Mice. Mol Cancer Ther.

[19] Migden MR, Rischin D, Schmults CD, Guminski A, Hauschild A, Lewis KD, Chung CH, Hernandez-Aya L, Lim AM, Chang ALS, Rabinowits G, Thai AA, Dunn LA, Hughes BGM, Khushalani NI, Modi B, Schadendorf D, Gao B, Seebach F, Li S, Li J, Mathias M, Booth J, Mohan K, Stankevich E, Babiker HM, Brana I, Gil-Martin M, Homsi J, Johnson ML, Moreno V, Niu J, Owonikoko TK, Papadopoulos KP, Yancopoulos GD, Lowy I, Fury MG (2018). PD-1 Blockade with Cemiplimab in Advanced Cutaneous Squamous-Cell Carcinoma. N Engl J Med.

[20] Owonikoko TK, Papadopoulos KP, Johnson ML, Gil Martín M, Moreno V, Salama AK, Calvo E, Yee NS, Safran H, Aljumaily R, Mahadevan D, Niu J, Kal Mohan K, Li J, Stankevich E, Mathias M, Lowy I, Fury MG, Babiker HM (2018). 71P - Phase I study of cemiplimab, a human monoclonal anti-PD-1, in patients with unresectable locally advanced or metastatic cutaneous squamous cell carcinoma (CSCC): Longer follow-up efficacy and safety data. Annals of Oncology.

[21] PharmaceuticalsInc R (2019) LIBTAYO® (cemiplimab-rwlc) full prescribing information (2019). https://www.regeneron.com/downloads/libtayo_fpi.pdf. Accessed April 15, 2021

[22] Migden MR, Chandra S, Rabinowits G, Chen CI, Desai J, Seluzhytsky A, Sasane M, Campanelli B, Chen Z, Freeman ML, Ibrahim SF, Khushalani NI, Andria M, Ruiz E (2020). CASE (CemiplimAb-rwlc Survivorship and Epidemiology) study in advanced cutaneous squamous cell carcinoma. Future Oncol.

[23] Rischin D, Migden MR, Lim AM, Schmults CD, Khushalani NI, Hughes BGM, Schadendorf D, Dunn LA, Hernandez-Aya L, Chang ALS, Modi B, Hauschild A, Ulrich C, Eigentler T, Stein B, Pavlick AC, Geiger JL, Gutzmer R, Alam M, Okoye E, Mathias M, Jankovic V, Stankevich E, Booth J, Li S, Lowy I, Fury MG, Guminski A (2020). Phase 2 study of cemiplimab in patients with metastatic cutaneous squamous cell carcinoma: primary analysis of fixed-dosing, long-term outcome of weight-based dosing. J Immunother Cancer.

[24] Rischin D, Fury MG, Lowy I, Stankevich E, Han H, Porceddu S (2020). A phase III, randomized, double-blind study of adjuvant cemiplimab versus placebo post-surgery and radiation therapy (RT) in patients (pts) with high-risk cutaneous squamous cell carcinoma (CSCC). Journal of Clinical Oncology.

[25] Sezer A, Kilickap S, Gümüş M, Bondarenko I, Özgüroğlu M, Gogishvili M, Turk HM, Cicin I, Bentsion D, Gladkov O, Clingan P, Sriuranpong V, Rizvi N, Gao B, Li S, Lee S, McGuire K, Chen CI, Makharadze T, Paydas S, Nechaeva M, Seebach F, Weinreich DM, Yancopoulos GD, Gullo G, Lowy I, Rietschel P (2021). Cemiplimab monotherapy for first-line treatment of advanced non-small-cell lung cancer with PD-L1 of at least 50%: a multicentre, open-label, global, phase 3, randomised, controlled trial. Lancet.

[26] Pharmaceuticals R (2021). https://investor.regeneron.com/index.php/news-releases/news-release-details/phase-3-trial-libtayor-cemiplimab-monotherapy-advanced-cervical.

[27] Reardon DA, Brem S, Desai AS, Bagley SJ, Kurz SC, Fuente MIDL, Nagpal S, Welch MR, Hormigo A, Carroll N, Bartra SK, Campbell P, Bhatt K, Lowy I, Boyer J, Kraynyak K, Morrow MP, McMullan T, Weiner DB, Skolnik J (2020). INO-5401 and INO-9012 delivered intramuscularly (IM) with electroporation (EP) in combination with cemiplimab (REGN2810) in newly diagnosed glioblastoma (GBM): Interim results. Journal of Clinical Oncology.

[28] Li R, Lee G, Huang M, El-Sherief A (2019). Rare basal cell metastasis of a basal-squamous skin collision tumour to the lung and axillary lymph node. BMJ Case Rep.

[29] Mycancergenome.org (2020) A Study of Recombinant Vaccinia Virus in Combination With Cemiplimab for Renal Cell Carcinoma. https://www.mycancergenome.org/content/clinical_trials/NCT03294083/. Accessed Apil 17, 2021

